# Supplementary material for: The ethical challenges of diversifying genomic data: A qualitative evidence synthesis
Source: Camb Prism Precis Med. 2023 Sep 12;2:e1. doi: 10.1017/pcm.2023.20 (PMC10953735; doi:10.1017/pcm.2023.20)
Supplement: Hardcastle et al. supplementary material [file S2752614323000200sup001.docx]

## Included studies (Rapid Review)

1. Abadie, Roberto, and Kathleen Heaney. 2015. ‘"We Can Wipe an Entire Culture’’: Fears and Promises of DNA Biobanking among Native Americans’. *DIALECTICAL ANTHROPOLOGY* 39 (3): 305–20.<https://doi.org/10.1007/s10624-015-9391-4>.
2. Ali-Khan, Sarah E., and Abdallah S. Daar. 2010. ‘Admixture Mapping: From Paradigms of Race and Ethnicity to Population History’. *The HUGO Journal* 4 (1–4): 23–34.<https://doi.org/10.1007/s11568-010-9145-y>.
3. Anie, Kofi A, Edeghonghon Olayemi, Vivian Paintsil, Ellis Owusu-Dabo, Titilope Adenike Adeyemo, Mahmoud U Sani, Najibah Aliyu Galadanci, et al. 2021. ‘Sickle Cell Disease Genomics of Africa (SickleGenAfrica) Network: Ethical Framework and Initial Qualitative Findings from Community Engagement in Ghana, Nigeria and Tanzania’. *BMJ Open* 11 (7): e048208.<https://doi.org/10.1136/bmjopen-2020-048208>.
4. Atkins, Rahshida, Terri-Ann Kelly, Shanda Johnson, Wanda Williams, Yolanda Nelson, Paule V. Joseph, Deirdre Jackson, et al. 2020. ‘Eliciting Willingness and Beliefs towards Participation in Genetic Psychiatric Testing in Black/African American Mothers at Risk for Depression’. *BEHAVIORAL SCIENCES* 10 (12).<https://doi.org/10.3390/bs10120181>.
5. Austin, M. A. 2002. ‘Ethical Issues in Human Genome Epidemiology: A Case Study Based on The Japanese American Family Study in Seattle, Washington’. *American Journal of Epidemiology* 155 (7): 585–92.<https://doi.org/10.1093/aje/155.7.585>.
6. Avera, Emily. 2009. ‘Rationalisation and Racialisation in the Rainbow Nation: Inequalities and Identity in the South African Bone Marrow Transplant Network’. *Anthropology & Medicine* 16 (2): 179–93.<https://doi.org/10.1080/13648470902940648>.
7. Balasopoulou, Angeliki, Foong-Ming Mooy, Darrol J. Baker, Christina Mitropoulou, Efthymios Skoufas, Awang Bulgiba, Theodora Katsila, and George P. Patrinos. 2017. ‘Advancing Global Precision Medicine: An Overview of Genomic Testing and Counseling Services in Malaysia’. *OMICS: A Journal of Integrative Biology* 21 (12): 733–40.<https://doi.org/10.1089/omi.2017.0136>.
8. Beans, Julie A., R. Brian Woodbury, Kyle A. Wark, Vanessa Y. Hiratsuka, and Paul Spicer. 2020. ‘Perspectives on Precision Medicine in a Tribally Managed Primary Care Setting’. *AJOB Empirical Bioethics* 11 (4): 246–56.<https://doi.org/10.1080/23294515.2020.1817172>.
9. Beaton A., Hudson M., Milne M., Port R.V., Russell K., Smith B., Toki V., et al. 2017. ‘Engaging Maori in Biobanking and Genomic Research: A Model for Biobanks to Guide Culturally Informed Governance, Operational, and Community Engagement Activities’. *Genetics in Medicine* 19 (3): 345–51.<https://doi.org/10.1038/gim.2016.111>.
10. Behring M., Hale K., Ozaydin B., Grizzle W.E., Sodeke S.O., and Manne U. 2019. ‘Inclusiveness and Ethical Considerations for Observational, Translational, and Clinical Cancer Health Disparity Research’. *Cancer* 125 (24): 4452–61.<https://doi.org/10.1002/cncr.32495>.
11. Bell, Hannah S., Funmi Odumosu, Anna C. Martinez-Hume, Heather A. Howard, and Linda M. Hunt. 2019. ‘Racialized Risk in Clinical Care: Clinician Vigilance and Patient Responsibility’. *MEDICAL ANTHROPOLOGY* 38 (3): 224–38.<https://doi.org/10.1080/01459740.2018.1476508>.
12. Blacksher, Erika, Vanessa Y. Hiratsuka, Jessica W. Blanchard, Justin R. Lund, Justin Reedy, Julie A. Beans, Bobby Saunkeah, et al. 2021. ‘Deliberations with American Indian and Alaska Native People about the Ethics of Genomics: An Adapted Model of Deliberation Used with Three Tribal Communities in the United States’. *AJOB Empirical Bioethics* 12 (3): 164–78.<https://doi.org/10.1080/23294515.2021.1925775>.
13. Bonham, Vence L., Toby Citrin, Stephen M. Modell, Tené Hamilton Franklin, Esther W. B. Bleicher, and Leonard M. Fleck. 2009. ‘Community-Based Dialogue: Engaging Communities of Color in the United States’ Genetics Policy Conversation’. *Journal of Health Politics, Policy and Law* 34 (3): 325–59.<https://doi.org/10.1215/03616878-2009-009>.
14. Buseh, Aaron G., Patricia E. Stevens, Sandra Millon-Underwood, Sheryl T. Kelber, and Leolia Townsend. 2017. ‘Embracing an “African Ethos” to Facilitate African Immigrants Participation in Medical Genetics and Genomics Research’. *NURSING OUTLOOK* 65 (1): 9–17.<https://doi.org/10.1016/j.outlook.2016.08.001>.
15. Buseh, Aaron G., Patricia E. Stevens, Sandra Millon-Underwood, Leolia Townsend, and Sheryl T. Kelber. 2013. ‘Community Leaders’ Perspectives on Engaging African Americans in Biobanks and Other Human Genetics Initiatives’. *Journal of Community Genetics* 4 (4): 483–94.<https://doi.org/10.1007/s12687-013-0155-z>.
16. Buseh, Aaron G., Sandra M. Underwood, Patricia E. Stevens, Leolia Townsend, and Sheryl T. Kelber. 2013. ‘Black African Immigrant Community Leaders’ Views on Participation in Genomics Research and DNA Biobanking’. *Nursing Outlook* 61 (4): 196–204.<https://doi.org/10.1016/j.outlook.2012.10.004>.
17. Campbell, Megan M., Ezra Susser, Sumaya Mall, Sibonile G. Mqulwana, Michael M. Mndini, Odwa A. Ntola, Mohamed Nagdee, Zukiswa Zingela, Stephanus Van Wyk, and Dan J. Stein. 2017. ‘Using Iterative Learning to Improve Understanding during the Informed Consent Process in a South African Psychiatric Genomics Study’. Edited by Bridget Young. *PLOS ONE* 12 (11): e0188466.<https://doi.org/10.1371/journal.pone.0188466>.
18. Canedo, Juan R., Consuelo H. Wilkins, Nicole Senft, Araceli Romero, Kemberlee Bonnet, and David Schlundt. 2020. ‘Barriers and Facilitators to Dissemination and Adoption of Precision Medicine among Hispanics/Latinos’. *BMC Public Health* 20 (1): 603.<https://doi.org/10.1186/s12889-020-08718-1>.
19. Chen, Lei-Shih, Mei Zhao, Qiong Zhou, and Lei Xu. 2012. ‘Chinese Americans’ Views of Prenatal Genetic Testing in the Genomic Era: A Qualitative Study’. *Clinical Genetics* 82 (1): 22–27.<https://doi.org/10.1111/j.1399-0004.2012.01871.x>.
20. Cohn, Elizabeth Gross, Maryam Husamudeen, Elaine L. Larson, and Janet K. Williams. 2015. ‘Increasing Participation in Genomic Research and Biobanking Through Community-Based Capacity Building’. *Journal of Genetic Counseling* 24 (3): 491–502.<https://doi.org/10.1007/s10897-014-9768-6>.
21. Culhane-Pera, Kathleen A., MaiKia Moua, Pachia Vue, Kang Xiaaj, May Xia Lo, and Robert J. Straka. 2017. ‘Leaves Imitate Trees: Minnesota Hmong Concepts of Heredity and Applications to Genomics Research’. *Journal of Community Genetics* 8 (1): 23–34.<https://doi.org/10.1007/s12687-016-0284-2>.
22. Culhane-Pera, Kathleen A., Robert J. Straka, MaiKia Moua, Youssef Roman, Pachia Vue, Kang Xiaaj, May Xia Lo, and Mai Lor. 2017. ‘Engaging Hmong Adults in Genomic and Pharmacogenomic Research: Toward Reducing Health Disparities in Genomic Knowledge Using a Community-Based Participatory Research Approach’. *Journal of Community Genetics* 8 (2): 117–25.<https://doi.org/10.1007/s12687-017-0292-x>.
23. De Ver Dye, Timothy, Zahira Quiñones Tavarez, José G Pérez Ramos, Isabel Diana Fernandez, Carmen Vélez Vega, Denisse M Vega Ocasio, Esteban Avendaño, et al. 2021. ‘Participation in Genetic Research among Latinx Populations by Latin America Birth-Residency Concordance: A Global Study’. *Journal of Community Genetics* 12 (4): 603–15.<https://doi.org/10.1007/s12687-021-00538-z>.
24. Dean, Caress, Amanda J. Fogleman, Whitney E. Zahnd, Alexander E. Lipka, Ripan Singh Malhi, Kristin R. Delfino, and Wiley D. Jenkins. 2017. ‘Engaging Rural Communities in Genetic Research: Challenges and Opportunities’. *Journal of Community Genetics* 8 (3): 209–19.<https://doi.org/10.1007/s12687-017-0304-x>.
25. Dirks, Lisa G., Jennifer L. Shaw, Vanessa Y. Hiratsuka, Julie A. Beans, Janet J. Kelly, and Denise A. Dillard. 2019. ‘Perspectives on Communication and Engagement with Regard to Collecting Biospecimens and Family Health Histories for Cancer Research in a Rural Alaska Native Community’. *Journal of Community Genetics* 10 (3): 435–46.<https://doi.org/10.1007/s12687-019-00408-9>.
26. Faure, Marlyn C., Olivia P. Matshabane, Patricia Marshall, Paul S. Appelbaum, Dan J. Stein, Mark E. Engel, and Jantina de Vries. 2019. ‘Does Genetics Matter for Disease-Related Stigma? The Impact of Genetic Attribution on Stigma Associated with Rheumatic Heart Disease in the Western Cape, South Africa’. *SOCIAL SCIENCE & MEDICINE* 243.<https://doi.org/10.1016/j.socscimed.2019.112619>.
27. Garofalo, Diana C., Howard A. Rosenblum, Yuan Zhang, Ying Chen, Paul S. Appelbaum, and Maya Sabatello. 2022. ‘Increasing Inclusivity in Precision Medicine Research: Views of Deaf and Hard of Hearing Individuals’. *Genetics in Medicine* 24 (3): 712–21.<https://doi.org/10.1016/j.gim.2021.11.015>.
28. Garrison, Nanibaa’ A., Krysta S. Barton, Kathryn M. Porter, Thyvu Mai, Wylie Burke, and Stephanie Russo Carroll. 2019. ‘Access and Management: Indigenous Perspectives on Genomic Data Sharing’. *Ethnicity & Disease* 29 (Supp): 659–68.<https://doi.org/10.18865/ed.29.S3.659>.
29. Godard, Béatrice, Vural Ozdemir, Marilyn Fortin, and Nathalie Égalité. 2010. ‘Ethnocultural Community Leaders’ Views and Perceptions on Biobanks and Population Specific Genomic Research: A Qualitative Research Study’. *Public Understanding of Science* 19 (4): 469–85.<https://doi.org/10.1177/0963662509104721>.
30. Goldenberg, Aaron J., Christopher D. Hartmann, Laura Morello, Sanjur Brooks, Kari Colón-Zimmermann, and Patricia A. Marshall. 2013. ‘Gene–Environment Interactions and Health Inequalities: Views of Underserved Communities’. *Journal of Community Genetics* 4 (4): 425–34.<https://doi.org/10.1007/s12687-013-0143-3>.
31. Goldsmith, Lesley, and Heather Skirton. 2015. ‘Research Involving People with a Learning Disability - Methodological Challenges and Ethical Considerations’. *JOURNAL OF RESEARCH IN NURSING* 20 (6): 435–46.<https://doi.org/10.1177/1744987115591867>.
32. Gordon, Elisa J., Daniela Amortegui, Isaac Blancas, Catherine Wicklund, John Friedewald, and Richard R. Sharp. 2019. ‘A Focus Group Study on African American Living Donors’ Treatment Preferences, Sociocultural Factors, and Health Beliefs About Apolipoprotein L1 Genetic Testing’. *PROGRESS IN TRANSPLANTATION* 29 (3): 239–47.<https://doi.org/10.1177/1526924819854485>.
33. Halbert, Chanita Hughes, Jasmine McDonald, Susan Vadaparampil, LaShanta Rice, and Melanie Jefferson. 2016. ‘Conducting Precision Medicine Research with African Americans’. Edited by Ornit Chiba-Falek. *PLOS ONE* 11 (7): e0154850.<https://doi.org/10.1371/journal.pone.0154850>.
34. Halverson, Colin M.E., and Lainie Friedman Ross. 2012. ‘Incidental Findings of Therapeutic Misconception in Biobank-Based Research’. *Genetics in Medicine* 14 (6): 611–15.<https://doi.org/10.1038/gim.2011.50>.
35. Haring, Rodney C., Whitney Ann Henry, Maui Hudson, Elisa M. Rodriguez, and Maile Taualii. 2018. ‘Views on Clinical Trial Recruitment, Biospecimen Collection, and Cancer Research: Population Science from Landscapes of the Haudenosaunee (People of the Longhouse)’. *Journal of Cancer Education* 33 (1): 44–51.<https://doi.org/10.1007/s13187-016-1067-5>.
36. Hendricks-Sturrup, Rachele M., and Tracey Johnson-Glover. 2021. ‘African American Nurses’ Perspectives on Genomic Medicine Research’. *AMA Journal of Ethics* 23 (3): E240-251.<https://doi.org/10.1001/amajethics.2021.240>.
37. Hiratsuka, Vanessa, Jennifer Brown, and Denise Dillard. 2012. ‘Views of Biobanking Research Among Alaska Native People: The Role of Community Context’. *Progress in Community Health Partnerships: Research, Education, and Action* 6 (2): 131–39.<https://doi.org/10.1353/cpr.2012.0025>.
38. Hiratsuka, Vanessa Y., Jennifer K. Brown, Theresa J. Hoeft, and Denise A. Dillard. 2012. ‘Alaska Native People’s Perceptions, Understandings, and Expectations for Research Involving Biological Specimens’. *International Journal Of Circumpolar Health* 71.<https://doi.org/10.3402/ijch.v71i0.18642>.
39. Hiratsuka, Vanessa Y., Michael J. Hahn, R. Brian Woodbury, Sara Chandros Hull, David R. Wilson, Vence L. Bonham, Denise A. Dillard, et al. 2020. ‘Alaska Native Genomic Research: Perspectives from Alaska Native Leaders, Federal Staff, and Biomedical Researchers’. *Genetics in Medicine* 22 (12): 1935–43.<https://doi.org/10.1038/s41436-020-0926-y>.
40. Hudson M., Beaton A., Milne M., Port W., Russell K., Smith B., Toki V., Uerata L., and Wilcox P. 2016. ‘Developing Models for Biobanking with Indigenous Peoples in New Zealand’. *Biopreservation and Biobanking* 14 (2): A5.<https://doi.org/10.1089/bio.2016.29001.abstracts>.
41. Hurlimann, Thierry, Iris Jaitovich Groisman, and Béatrice Godard. 2017. ‘The Elusive Ideal of Inclusiveness: Lessons from a Worldwide Survey of Neurologists on the Ethical Issues Raised by Whole-Genome Sequencing’. *BMC Medical Ethics* 18 (1): 28.<https://doi.org/10.1186/s12910-017-0187-8>.
42. Hutchinson, Janis Faye, and Richard Sharp. 2008. ‘Karma, Reincarnation, and Medicine: Hindu Perspectives on Biomedical Research.’ *Genomic Medicine* 2 (3–4): 107–11.<https://doi.org/10.1007/s11568-009-9079-4>.
43. Igbe, Michael A, and Clement A Adebamowo. 2012. ‘Qualitative Study of Knowledge and Attitudes to Biobanking among Lay Persons in Nigeria’. *BMC Medical Ethics* 13 (1): 27.<https://doi.org/10.1186/1472-6939-13-27>.
44. Jacobs, Bette, Jason Roffenbender, Jeff Collmann, Kate Cherry, LeManuel Lee Bitsói, Kim Bassett, and Charles H. Evans. 2010. ‘Bridging the Divide between Genomic Science and Indigenous Peoples’. *Journal of Law, Medicine & Ethics* 38 (3): 684–96.<https://doi.org/10.1111/j.1748-720X.2010.00521.x>.
45. Kaplan, Benjamin, Carolyn Caddle-Steele, Gregory Chisholm, Warria A. Esmond, Kadija Ferryman, Melvin Gertner, Crispin Goytia, et al. 2017. ‘A Culture of Understanding: Reflections and Suggestions from a Genomics Research Community Board’. *PROGRESS IN COMMUNITY HEALTH PARTNERSHIPS-RESEARCH EDUCATION AND ACTION* 11 (2): 161–65.<https://doi.org/10.1353/cpr.2017.0020>.
46. Kobayashi, Yoko, Patrick Boudreault, Karin Hill, Janet S. Sinsheimer, and Christina G. S. Palmer. 2013. ‘Using a Social Marketing Framework to Evaluate Recruitment of a Prospective Study of Genetic Counseling and Testing for the Deaf Community’. *BMC MEDICAL RESEARCH METHODOLOGY* 13.<https://doi.org/10.1186/1471-2288-13-145>.
47. Kowal, Emma. 2013. ‘Orphan DNA: Indigenous Samples, Ethical Biovalue and Postcolonial Science’. *SOCIAL STUDIES OF SCIENCE* 43 (4): 577–97.<https://doi.org/10.1177/0306312712471739>.
48. Kowal Emma. 2019. ‘Indigenous People and Genomics in Australia’. *Twin Research and Human Genetics* 22 (5): 332.<https://doi.org/10.1017/thg.2019.80>.
49. Kraft, Stephanie A., Mildred K. Cho, Katherine Gillespie, Meghan Halley, Nina Varsava, Kelly E. Ormond, Harold S. Luft, Benjamin S. Wilfond, and Sandra Soo-Jin Lee. 2018. ‘Beyond Consent: Building Trusting Relationships With Diverse Populations in Precision Medicine Research’. *The American Journal of Bioethics* 18 (4): 3–20.<https://doi.org/10.1080/15265161.2018.1431322>.
50. Kraft, Stephanie A., and Megan Doerr. 2018. ‘Engaging Populations Underrepresented in Research through Novel Approaches to Consent’. *American Journal of Medical Genetics Part C: Seminars in Medical Genetics* 178 (1): 75–80.<https://doi.org/10.1002/ajmg.c.31600>.
51. Kraft, Stephanie A., Carmit McMullen, Nangel M. Lindberg, David Bui, Kelly Shipman, Katherine Anderson, Galen Joseph, et al. 2020. ‘Integrating Stakeholder Feedback in Translational Genomics Research: An Ethnographic Analysis of a Study Protocol’s Evolution’. *Genetics in Medicine* 22 (6): 1094–1101.<https://doi.org/10.1038/s41436-020-0763-z>.
52. Kraft, Stephanie A, Erin Rothwell, Seema K Shah, Devan M Duenas, Hannah Lewis, Kristin Muessig, Douglas J Opel, Katrina A B Goddard, and Benjamin S Wilfond. 2021. ‘Demonstrating “Respect for Persons” in Clinical Research: Findings from Qualitative Interviews with Diverse Genomics Research Participants’. *Journal of Medical Ethics* 47 (12): e8–e8.<https://doi.org/10.1136/medethics-2020-106440>.
53. Lee, Sandra S.-J., Mildred K. Cho, Stephanie A. Kraft, Nina Varsava, Katie Gillespie, Kelly E. Ormond, Benjamin S. Wilfond, and David Magnus. 2019. ‘“I Don’t Want to Be Henrietta Lacks”: Diverse Patient Perspectives on Donating Biospecimens for Precision Medicine Research’. *Genetics in Medicine* 21 (1): 107–13.<https://doi.org/10.1038/s41436-018-0032-6>.
54. Lehmann, Lisa Soleymani, Jane C. Weeks, Neil Klar, and Judy E. Garber. 2002. ‘A Population-Based Study of Ashkenazi Jewish Women’s Attitudes toward Genetic Discrimination and BRCA1/2 Testing’. *Genetics in Medicine* 4 (5): 346–52.<https://doi.org/10.1097/00125817-200209000-00005>.
55. Lewis, Katie L., Alexis R. Heidlebaugh, Sandra Epps, Paul K.J. Han, Kristen P. Fishler, William M.P. Klein, Ilana M. Miller, et al. 2019. ‘Knowledge, Motivations, Expectations, and Traits of an African, African-American, and Afro-Caribbean Sequencing Cohort and Comparisons to the Original ClinSeq® Cohort’. *Genetics in Medicine* 21 (6): 1355–62.<https://doi.org/10.1038/s41436-018-0341-9>.
56. Lewis-Fernández, Roberto, Angela A. Coombs, Iván C. Balán, and Alejandro Interian. 2018. ‘Motivational Interviewing: Overcoming Disparities in Pharmacotherapy Engagement’. *The Journal of Clinical Psychiatry* 79 (3).<https://doi.org/10.4088/JCP.18ac12150>.
57. Llanos, Adana A.M., Gregory S. Young, Ryan Baltic, Eugene J. Lengerich, Betsy B. Aumiller, Mark B. Dignan, and Electra D. Paskett. 2018. ‘Predictors of Willingness to Participate in Biospecimen Donation and Biobanking among Appalachian Adults’. *Journal of Health Care for the Poor and Underserved* 29 (2): 743–66.<https://doi.org/10.1353/hpu.2018.0056>.
58. Lysaght, Tamra, Angela Ballantyne, Vicki Xafis, Serene Ong, Gerald Owen Schaefer, Jeffrey Min Than Ling, Ainsley J. Newson, Ing Wei Khor, and E. Shyong Tai. 2020. ‘“Who Is Watching the Watchdog?”: Ethical Perspectives of Sharing Health-Related Data for Precision Medicine in Singapore’. *BMC Medical Ethics* 21 (1): 118.<https://doi.org/10.1186/s12910-020-00561-8>.
59. Marsh, Vicki, Francis Kombe, Raymond Fitzpatrick, Thomas N Williams, Michael Parker, and Sassy Molyneux. 2013. ‘Consulting Communities on Feedback of Genetic Findings in International Health Research: Sharing Sickle Cell Disease and Carrier Information in Coastal Kenya’. *BMC Medical Ethics* 14 (1): 41.<https://doi.org/10.1186/1472-6939-14-41>.
60. Marshall, Patricia A, Clement A Adebamowo, Adebowale A Adeyemo, Temidayo O Ogundiran, Teri Strenski, Jie Zhou, and Charles N Rotimi. 2014. ‘Voluntary Participation and Comprehension of Informed Consent in a Genetic Epidemiological Study of Breast Cancer in Nigeria’. *BMC Medical Ethics* 15 (1): 38.<https://doi.org/10.1186/1472-6939-15-38>.
61. Matimba, Alice, Gunnel Tybring, Jekoniya Chitereka, Rutendo Zinyama-Gutsire, Collet Dandara, Eva Bürén, Milcah Dhoro, and Collen Masimirembwa. 2016. ‘Practical Approach to Biobanking in Zimbabwe: Establishment of an Inclusive Stakeholder Framework’. *Biopreservation and Biobanking* 14 (5): 440–46.<https://doi.org/10.1089/bio.2015.0043>.
62. Mulder, Nicola, Alash’le Abimiku, Sally N Adebamowo, Jantina de Vries, Alice Matimba, Paul Olowoyo, Michele Ramsay, Michelle Skelton, and Dan J Stein. 2018. ‘H3Africa: Current Perspectives’. *Pharmacogenomics and Personalized Medicine* Volume 11 (April): 59–66.<https://doi.org/10.2147/PGPM.S141546>.
63. Munung, N. S., B. M. Mayosi, and J. de Vries. 2018. ‘Genomics Research in Africa and Its Impact on Global Health: Insights from African Researchers’. *Global Health, Epidemiology and Genomics* 3: e12.<https://doi.org/10.1017/gheg.2018.3>.
64. Neidich A.B., Joseph J.W., Ober C., and Ross L.F. 2008. ‘Empirical Data about Women’s Attitudes towards a Hypothetical Pediatric Biobank’. *American Journal of Medical Genetics, Part A* 146 (3): 297–304.<https://doi.org/10.1002/ajmg.a.32145>.
65. Nembaware, Victoria, Katherine Johnston, Alpha A. Diallo, Maritha J. Kotze, Alice Matimba, Keymanthri Moodley, Godfrey B. Tangwa, Rispah Torrorey-Sawe, and Nicki Tiffin. 2019. ‘A Framework for Tiered Informed Consent for Health Genomic Research in Africa’. *NATURE GENETICS* 51 (11): 1566–71.<https://doi.org/10.1038/s41588-019-0520-x>.
66. Norstad, Matthew, Simon Outram, Julia E.H. Brown, Astrid N. Zamora, Barbara A. Koenig, Neil Risch, Mary E. Norton, Anne Slavotinek, and Sara L. Ackerman. 2022. ‘The Difficulties of Broad Data Sharing in Genomic Medicine: Empirical Evidence from Diverse Participants in Prenatal and Pediatric Clinical Genomics Research’. *Genetics in Medicine* 24 (2): 410–18.<https://doi.org/10.1016/j.gim.2021.09.021>.
67. Nwulia, E. A., M. M. Hipolito, S. Aamir, W. B. Lawson, and J. I. Nurnberger Jr. 2011. ‘Ethnic Disparities in the Perception of Ethical Risks From Psychiatric Genetic Studies’. *AMERICAN JOURNAL OF MEDICAL GENETICS PART B-NEUROPSYCHIATRIC GENETICS*, BIGS Consortium, 156B (5): 569–80.<https://doi.org/10.1002/ajmg.b.31198>.
68. Ogunrin, Olubunmi, Mark Gabbay, Kerry Woolfall, and Lucy Frith. 2022. ‘Community Engagement in Genomic Research: Proposing a Strategic Model for Effective Participation of Indigenous Communities’. *Developing World Bioethics* 22 (4): 189–202.<https://doi.org/10.1111/dewb.12329>.
69. Oushy, Mai H., Rebecca Palacios, Alan E. C. Holden, Amelie G. Ramirez, Kipling J. Gallion, and Mary A. O’Connell. 2015. ‘To Share or Not to Share? A Survey of Biomedical Researchers in the US Southwest, an Ethnically Diverse Region’. *PLOS ONE* 10 (9).<https://doi.org/10.1371/journal.pone.0138239>.
70. Panofsky, Aaron. 2019. ‘Scientific Racism: From Dissident Professionals to Citizen Science’. In *American Journal of Physical Anthropology*, 168:185.<https://doi.org/10.1002/ajpa.23802>.
71. Reddy, Arthi, Abhimanyu Amarnani, Michael Chen, Sophia Dynes, Bryan Flores, Ariella Moshchinsky, Yeon Joo Lee, et al. 2020. ‘Privacy Concerns About Personal Health Information and Fear of Unintended Use of Biospecimens Impact Donations by African American Patients’. *Journal of Cancer Education* 35 (3): 522–29.<https://doi.org/10.1007/s13187-019-01491-9>.
72. Ridgeway, Jennifer L., Monica Albertie, Elizabeth Pantoja, Darin Prescott, Xuan Zhu, and Carmen Radecki Breitkopf. 2019. ‘Understanding Diverse Perspectives on Genetic Research Through Focus Group Talk’. *INTERNATIONAL JOURNAL OF QUALITATIVE METHODS* 18.<https://doi.org/10.1177/1609406919892476>.
73. Rosas, Lisa G., Catherine Nasrallah, Van Ta Park, Jan J. Vasquez, Ysabel Duron, Owen Garrick, Riccesha Hattin, et al. 2020. ‘Perspectives on Precision Health Among Racial/Ethnic Minority Communities and the Physicians That Serve Them’. *Ethnicity & Disease* 30 (Suppl 1): 137–48.<https://doi.org/10.18865/ed.30.S1.137>.
74. Sabatello, Maya, Lou Ann Blake, Audrey Chao, Arielle Silverman, Ronit Ovadia Mazzoni, Yuan Zhang, Ying Chen, and Paul S. Appelbaum. 2019. ‘Including the Blind Community in Precision Medicine Research: Findings from a National Survey and Recommendations’. *Genetics in Medicine: Official Journal of the American College of Medical Genetics* 21 (11): 2631–38.<https://doi.org/10.1038/s41436-019-0533-y>.
75. Sabatello, Maya, Ying Chen, Yuan Zhang, and Paul S. Appelbaum. 2019. ‘Disability Inclusion in Precision Medicine Research: A First National Survey’. *Genetics in Medicine: Official Journal of the American College of Medical Genetics* 21 (10): 2319–27.<https://doi.org/10.1038/s41436-019-0486-1>.
76. Saulsberry, Loren, Keith Danahey, Brittany A. Borden, Elizabeth Lipschultz, Maimouna Traore, Mark J. Ratain, David O. Meltzer, and Peter H. O’Donnell. 2021. ‘Underrepresented Patient Views and Perceptions of Personalized Medication Treatment through Pharmacogenomics’. *Npj Genomic Medicine* 6 (1): 90.<https://doi.org/10.1038/s41525-021-00253-1>.
77. Savich, Renate D., Beth B Tigges, Lisbeth Iglesias Rios, Joanne McCloskey, Kristine Tollestrup, and Robert D. Annett. 2020. ‘Willingness of Women to Participate in Obstetrical and Pediatric Research Involving Biobanks’. *Journal of Community Genetics* 11 (2): 215–23.<https://doi.org/10.1007/s12687-019-00446-3>.
78. Schulz, Amy, Cleopatra Caldwell, and Sarah Foster. 2003. ‘“What Are They Going to Do With the Information?” Latino/Latina and African American Perspectives on the Human Genome Project’. *Health Education & Behavior* 30 (2): 151–69.<https://doi.org/10.1177/1090198102251026>.
79. Scott, Denise M., Veronica G. Thomas, Jane Otado, Faun Rockcliffe, Omotomilade Olopoenia, Dietrich Johnson, and Shawneequa Callier. 2020. ‘Attitudes and Experiences Regarding Genetic Research among Persons of African Descent’. *Journal of Community Genetics* 11 (1): 65–72.<https://doi.org/10.1007/s12687-019-00422-x>.
80. Shaibi, Gabriel Q., Dawn K. Coletta, Veronica Vital, and Lawrence J. Mandarino. 2013. ‘The Design and Conduct of a Community-Based Registry and Biorepository: A Focus on Cardiometabolic Health in Latinos’. *CTS-CLINICAL AND TRANSLATIONAL SCIENCE* 6 (6): 429–34.<https://doi.org/10.1111/cts.12114>.
81. Shaibi, Gabriel Q., Iftikhar J. Kullo, Davinder P. Singh, Richard R. Sharp, Eleanna De Filippis, Idali Cuellar, Valentina Hernandez, et al. 2018. ‘Developing a Process for Returning Medically Actionable Genomic Variants to Latino Patients in a Federally Qualified Health Center’. *Public Health Genomics* 21 (1–2): 77–84.<https://doi.org/10.1159/000494488>.
82. Sheppard, Vanessa B., Darren Mays, Kenneth P. Tercyak, and Thomas LaVeist. 2013. ‘Medical Mistrust Influences Black Women’s Level of Engagement in BRCA1/2 Genetic Counseling and Testing’. *Journal of the National Medical Association* 105 (1): 17–22.<https://doi.org/10.1016/S0027-9684(15)30081-X>.
83. Shields A.E., Burke W., and Levy D.E. 2008. ‘Differential Use of Available Genetic Tests among Primary Care Physicians in the United States: Results of a National Survey’. *Genetics in Medicine* 10 (6): 404–14.<https://doi.org/10.1097/GIM.0b013e3181770184>.
84. Simon, Melissa A., Erika E. de la Riva, Raymond Bergan, Carrie Norbeck, June M. McKoy, Piotr Kulesza, XinQi Dong, Julian Schink, and Linda Fleisher. 2014. ‘Improving Diversity in Cancer Research Trials: The Story of the Cancer Disparities Research Network’. *Journal of Cancer Education* 29 (2): 366–74.<https://doi.org/10.1007/s13187-014-0617-y>.
85. Simon, Melissa A., Laura S. Tom, and XinQi Dong. 2017. ‘Knowledge and Beliefs About Biospecimen Research Among Chinese Older Women in Chicago’s Chinatown’. *JOURNALS OF GERONTOLOGY SERIES A-BIOLOGICAL SCIENCES AND MEDICAL SCIENCES* 72: S41–49.<https://doi.org/10.1093/gerona/glw333>.
86. Sinclair, Ka’imi A., Clemma Muller, Carolyn Noonan, Cathryn Booth-LaForce, and Dedra S. Buchwald. 2021. ‘Increasing Health Equity through Biospecimen Research: Identification of Factors That Influence Willingness of Native Americans to Donate Biospecimens’. *Preventive Medicine Reports* 21 (March): 101311.<https://doi.org/10.1016/j.pmedr.2021.101311>.
87. Singh, Sachil, and Valerie Steeves. 2020. ‘The Contested Meanings of Race and Ethnicity in Medical Research: A Case Study of the DynaMed Point of Care Tool’. *Social Science & Medicine* 265 (November): 113112.<https://doi.org/10.1016/j.socscimed.2020.113112>.
88. Storr, Carla L., Flora Or, William W. Eaton, and Nicholas Ialongo. 2014. ‘Genetic Research Participation in a Young Adult Community Sample’. *Journal of Community Genetics* 5 (4): 363–75.<https://doi.org/10.1007/s12687-014-0191-3>.
89. Suther, Sandra, and Gebre-Egziabher Kiros. 2009. ‘Barriers to the Use of Genetic Testing: A Study of Racial and Ethnic Disparities’. *Genetics in Medicine* 11 (9): 655–62.<https://doi.org/10.1097/GIM.0b013e3181ab22aa>.
90. Svalastog, Anna Lydia. 2013. ‘Making It Transparent. On Naming, Framing and Administrating Biobank Research on Native People’. *New Genetics and Society* 32 (3): 209–42.<https://doi.org/10.1080/14636778.2012.760265>.
91. Ta Park V., Kim A., Cho I.H., Nam B., Nguyen K., Vuong Q., Periyakoil V.S., and Hong Y.A. 2021. ‘Motivation to Participate in Precision Health Research and Acceptability of Texting as a Recruitment and Intervention Strategy Among Vietnamese Americans: Qualitative Study’. *JMIR MHealth and UHealth* 9 (3): e23058.<https://doi.org/10.2196/23058>.
92. Taualii, Maile, Elise Leimomi Davis, Kathryn L. Braun, JoAnn Umilani Tsark, Ngiare Brown, Maui Hudson, and Wylie Burke. 2014. ‘Native Hawaiian Views on Biobanking’. *JOURNAL OF CANCER EDUCATION* 29 (3): 570–76.<https://doi.org/10.1007/s13187-014-0638-6>.
93. Trinidad S.B., Blacksher E., Woodbury R.B., Hopkins S.E., Burke W., Woodahl E.L., Boyer B.B., and Hiratsuka V.Y. 2022. ‘Precision Medicine Research with American Indian and Alaska Native Communities: Results of a Deliberative Engagement with Tribal Leaders’. *Genetics in Medicine*, no. (Trinidad, Blacksher, Burke) Department of Bioethics and Humanities, UW School of Medicine, Seattle, WA, United States(Woodbury, Hiratsuka) Research Department, Southcentral Foundation, Anchorage, AK, United States(Hopkins, Boyer) Department of Obstetrics.<https://doi.org/10.1016/j.gim.2021.11.003>.
94. Vries, Jantina de, Muminatou Jallow, Thomas N. Williams, Dominic Kwiatkowski, Michael Parker, and Raymond Fitzpatrick. 2012. ‘Investigating the Potential for Ethnic Group Harm in Collaborative Genomics Research in Africa: Is Ethnic Stigmatisation Likely?’ *Social Science & Medicine* 75 (8): 1400–1407.<https://doi.org/10.1016/j.socscimed.2012.05.020>.
95. Vries, Jantina de, Thomas N Williams, Kalifa Bojang, Dominic P Kwiatkowski, Raymond Fitzpatrick, and Michael Parker. 2014. ‘Knowing Who to Trust: Exploring the Role of “Ethical Metadata” in Mediating Risk of Harm in Collaborative Genomics Research in Africa’. *BMC Medical Ethics* 15 (1): 62.<https://doi.org/10.1186/1472-6939-15-62>.
96. Walker, Evelyn R., Cheryl R. Nelson, Donna Antoine-LaVigne, Darcel T. Thigpen, Mona A. Puggal, Daniel E. Sarpong, and Alice M. Smith. 2014. ‘Research Participants’ Opinions on Genetic Research and Reasons for Participation: A Jackson Heart Study Focus Group Analysis’. *Ethnicity & Disease* 24 (3): 290–97.
97. Winickoff David E. 2003. ‘Governing Population Genomics: Law, Bioethics, and Biopolitics in Three Case Studies’. *Jurimetrics* 43 (2): 187–228.<https://www.jstor.org/stable/29762805>.
98. Woof, Victoria G., Helen Ruane, David P. French, Fiona Ulph, Nadeem Qureshi, Nasaim Khan, D. Gareth Evans, and Louise S. Donnelly. 2020. ‘The Introduction of Risk Stratified Screening into the NHS Breast Screening Programme: Views from British-Pakistani Women’. *BMC Cancer* 20 (1): 452.<https://doi.org/10.1186/s12885-020-06959-2>.
99. Wright, Galen EB, Pieter GJ Koornhof, Adebowale A. Adeyemo, and Nicki Tiffin. 2013. ‘Ethical and Legal Implications of Whole Genome and Whole Exome Sequencing in African Populations’. *BMC Medical Ethics* 14 (1): 21.<https://doi.org/10.1186/1472-6939-14-21>.
100. Yeh, Vivian M., Erin M. Bergner, Marino A. Bruce, Sunil Kripalani, Victoria B. Mitrani, Titilola A. Ogunsola, Consuelo H. Wilkins, and Derek M. Griffith. n.d. ‘Can Precision Medicine Actually Help People Like Me? African American and Hispanic Perspectives on the Benefits and Barriers of Precision Medicine’. *Ethnicity & Disease* 30 (Suppl 1): 149–58. Accessed 3 August 2022.<https://doi.org/10.18865/ed.30.S1.149>.
